# Analysis of oral yeast microflora in patients with oral squamous cell carcinoma

**DOI:** 10.1186/s40064-016-2926-6

**Published:** 2016-08-04

**Authors:** Csaba Berkovits, Adél Tóth, Judit Szenzenstein, Tünde Deák, Edit Urbán, Attila Gácser, Katalin Nagy

**Affiliations:** 1 Department of Oral Surgery, University of Szeged, Tisza Lajos krt. 64, Szeged, 6720 Hungary; 2 Department of Microbiology, University of Szeged, Közép fasor 52, Szeged, 6726 Hungary; 3 Institute of Clinical Microbiology, University of Szeged, Semmelweis u. 6, Szeged, 6725 Hungary

**Keywords:** Oral squamous cell carcinoma, MALDI-TOF, *Candida*, Yeast

## Abstract

**Background:**

Oral squamous cell carcinoma (OSCC) is the most common form of oral cancer, in this study, the association between OSCC and oral yeast carriage was investigated.

**Findings:**

20 patients having OSCC as well as 40 healthy controls were tested for the presence of yeasts in the oral cavity. Fungal burdens were examined by colony forming unit determinations, while the different yeast genera in patient samples were identified by matrix-associated laser desorption/ionization-time of flight-mass spectrometry. We found that the level of oral yeast carriage was significantly higher in patients with OSCC that was accompanied by a higher diversity of yeasts in the oral cavity of these patients. We also examined the extracellular enzyme production of isolated *Candida* spp.; however, we found that there was no association between the lipase/protease producing capacity of *Candida* strains and the higher colonisation rate of neoplastic epithelium.

**Conclusions:**

In conclusion, our results corroborate the findings of previous studies regarding the association between oral yeast carriage and epithelial carcinoma.

## Background

Oral squamous cell carcinoma (OSCC) is the most common form of oral cancer, representing up to 80–90 % of all malignancies of the oral cavity (Pires et al. 2013). Among others, risk factors for OSCC include smoking, alcohol consumption, ultraviolet radiation and poor oral hygiene (Mohd et al. 2010). The occurrence of OSCC has also been associated with *Candida* infections, although the underlying pathogenic mechanisms are poorly understood (Mohd et al. 2010).

*Candida* species belong to the normal flora and are frequently isolated from various mucosal surfaces in healthy individuals (Sardi et al. 2013). However, they may cause cutaneous or systemic infections when the immunity of the host is compromised. Furthermore, although little is known about the role of fungal infections in cancer, *Candida* spp. have long been implicated in various epithelial malignancies. There are several reports about chronic mucocutaneous candidiasis (CMC) patients developing oral or esophageal carcinoma (Mohd et al. 2010). Furthermore, chronic hyperplastic candidiasis (CHC, candidal leukoplakia), a rare form of oral candidiasis has been shown to often undergo malignant transformation (Cawson 1966). A recent study has found that *Candida* species can be isolated with higher frequency from patients with oral epithelial dysplasia compared to healthy subjects (Hebbar and Pai 2013). *Candida* spp. have several attributes that may promote oral cancer development, such as the ability to produce carcinogens (e.g. nitrosamines), metabolize procarcinogens or induce inflammation (Mohd et al. 2010). However, the etiological relationship between oral cancer and *Candida* infections is still a matter of debate and needs to be further investigated.

In this study, we assessed the presence of *Candida* spp. in the oral cavity of patients having keratinizing OSCC as well as healthy controls to investigate the association between *Candida* infections and oral cancer development.

## Methods

### Patients

A total of 60 subjects [20 OSCC patients (14 males, 6 females, median age: 62 (61.95), range 44–86) and 40 controls (22 males, 18 females, median age: 67 (67.62), range 49–82)] were enrolled in this study. The patients and the controls were recruited from among the patients of the Departments of Dentoalveolar Surgery and Maxillofacial Surgery at the Faculties of Dentistry and Medicine at the University of Szeged. OSCC patients were eligible for this study if they had a histologically confirmed diagnosis and if they had not received any treatment for OSCC up to their participation. Controls were recruited from outpatients free of oral mucosal pathology who arrived for routine procedures (e.g. tooth extraction). The study design complied with the tenets of the Declaration of Helsinki in all respects, and it was approved by the Research Ethics Committee for Human Medical Biology at the University of Szeged. The participation was voluntary and it was based on informed consent. Before their enrolment, all subjects received information about the background, aims and procedures of the study, and they had to sign an informed consent form to signify that they opted for participation by their own free will and based on the information they got. Frequency and locations of cancer sites in oral cancer patients: T1: carcinoma fundi oris l.d., T2: carcinoma linguae l.d., T3: carcinoma hypopharyngis l.s., T4: carcinoma radicis linguae l.d., T5. carcinoma gingivae l.s., T6: carcinoma hypopharyngis l.s., T7: carcinoma linguae l.d, T8: carcinoma fundi oris l.s., T9: carcinoma fundi oris l.d., T10: carcinoma linguae l.s., T11: carcinoma radicis linguae l.s., T12: carcinoma linguae l.s., T13: carcinoma labii superioris vestibularis l.d., T14: carcinoma labii inferioris vestibularis l.s., T15: carcinoma mandibulae ging. l.d., T16: carcinoma labii inferioris vestibularis l.d., T17: carcinoma fundi oris et gingivae l.s., T18: carcinoma linguae l.d., T19: carcinoma fundi oris l.d., T20: carcinoma hypopharyngis l.d.

### Oral samples

Oral swabs were taken from a 1 cm^2^ area from two different locations in the oral cavity (in the case of OSCC patients, both from the surface of neoplastic and healthy epithelium). Samples were inoculated on Sabouraud dextrose agar plates and Sabouraud broth, both incubated for 7 days at room atmosphere, in 32 °C. Direct samples that tested negative for yeast growth on agar plates after the first 3 days were subcultured from the liquid medium and incubated again for 7 days (referred to as “subculture”). Basic identification of the isolated yeasts was carried out based on macro- and micro-scopic morphology, catalase test and CHROMagar Candida plates (Becton–Dickinson, UK), next MALDI-TOF (Bruker Daltonics, Bremen, Gr) analysis was carried out from all of the isolates.

### MALDI-TOF-MS

Sample preparation was carried out according to the Bruker protocols using three methods: (1) direct transfer (DT), (2) extended direct transfer (eDT), and (3) ethanol (EtOH)–formic acid (FA) extraction. In the DT method, a thin smear of biological material was placed onto a target plate, which was immediately overlaid with 1 μl of alpha-cyano-4-hydroxycinnamic acid matrix solution (HCCA; Bruker Daltonics, Gr.), prepared according to the protocol of the manufacturer. In the eDT method, the biomass was treated with 1 μl of 70 % FA on the target plate prior to the HCCA matrix overlay. In the EtOH–FA method, one or two loops of yeast biomass (1 μl volume, sterile inoculation loop) was used for the crude protein extraction, as described previously (Lacroix et al. 2014). One microliter of the crude protein extract was spotted onto the target plate, and after air-drying, it was overlaid with 1 μl of HCCA matrix solution. For all methods, each tested strain was spotted in duplicate. Standard commercially available Bruker Daltonics database (BDAL) was used in this study: individual spectra using the MALDI Biotyper automated FlexControl software version 3.0 (Bruker Daltonics). The MALDI-TOF MS identification results were automatically classified using the log-score values generated by the MALDI Biotyper software (Bruker Daltonics, Germany), performed according to the manufacturer’s instructions. Scores higher than 1.7 indicated genus-level identification.

### Analysis of lipase and protease activity

Extracellular lipase activity of *Candida* strains was examined on YNB-rhodamine B plates as previously described (Nemeth et al. 2013). Briefly, strains were inoculated onto YNB-rhodamine B (yeast nitrogen base) plates (Sigma-Aldrich) and incubated at 30 °C for 7 days. Lipase positivity was determined by the presence of pink halo around the colonies. For the detection of proteolytic activity, *Candida* strains were cultured on YCB-BSA (yeast carbon base—bovine serum albumin, Sigma-Aldrich) agar plates at 30 °C for 7 days, and proteolysis around the colonies was visualised by amido black (Sigma-Aldrich) staining (Nemeth et al. 2013).

### Statistical analysis

All statistical analyses have been performed by GraphPad Prism 5.0 software. Fisher’s exact test, Mann–Whitney test or Wilcoxon signed-rank test were used wherever appropriate. Differences between groups were considered significant at *p* < 0.05.

## Results

**Fig. 1:**
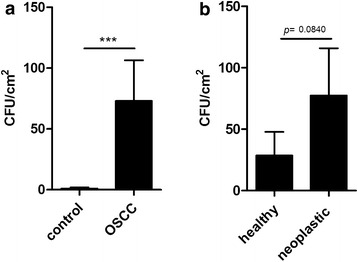
Fungal burdens in the oral cavity of OSCC patients and healthy controls. **a** Average fungal burdens (mean ± SEM) in the oral cavity of control (n = 40) and OSCC patients (n = 20). In the case of OSCC patients, the average fungal burden of healthy and neoplastic epithelial surface is shown. ***p < 0.001 (Mann–Whitney test). **b** Average fungal burdens on the healthy and neoplastic epithelial surface in the oral cavity of OSCC patients (n = 20). p value was determined by Wilcoxon signed-rank test

20 patients with confirmed OSCC as well as 40 healthy controls were enrolled in the study. 18 (90 %) of the 20 OSCC patients and 12 (30 %) of the healthy controls had yeast isolated from their oral cavity (Table 1), indicating that the frequency of oral yeast colonization was significantly higher in OSCC patients compared to the control group (Fisher’s exact test, *p* < 0.0001). Furthermore, OSCC patients had a significantly higher average fungal burden (mean ± SEM, 73.08 ± 33.39 CFU/cm^2^) in their oral cavity compared to healthy individuals (1.10 ± 0.78 CFU/cm^2^, Fig. 1a), and samples taken from the neoplastic surface contained more yeast cells (77.38 ± 38.53 CFU/cm^2^) compared to the swabs taken from the healthy epithelium of the same individual (28.58 ± 19.18 CFU/cm^2^, Fig. 1b). To assess the spectrum of yeast genera present in the oral cavity of healthy or OSCC patients, samples were subjected to peptide mass fingerprinting by matrix-associated laser desorption/ionization-time of flight-mass spectrometry (MALDI-TOF-MS). While the predominant fungal genus present in the oral cavity of both healthy and OSCC patients was *Candida*, we found that there was a higher diversity of yeasts in the oral swabs of cancer patients compared to healthy controls (Table 2). As the secretion of hydrolytic enzymes is an important virulence factor of *Candida* spp. (Nemeth et al. 2013; Stehr et al. 2004; Trofa et al. 2009; Monod and Borg-von 2002), we also examined whether the higher colonisation rate in the oral cavities of OSCC patients is associated with an increased prevalence of *Candida* strains with hydrolytic enzyme activity. To analyse the enzyme profiles of *Candida* strains, we have randomly chosen 40 isolates from healthy controls and 140 isolates from OSCC patients and tested their extracellular lipase and protease activity. However, we found that the enzyme production of isolates derived from the two different patient group did not differ significantly (Table 3).

**Table 1:** Prevalence of oral yeast carriage in OSCC patients and healthy controls

Oral yeast carriage	Positive, *N* (%)	Negative, *N* (%)
Control patients (n = 40)		
Original sample	3 (7.5)	37 (92.5)
Subculture	12 (30)	28 (70)
OSCC patients (n = 20)		
Original sample	10 (50)	10 (50)
Subculture	18 (90)	2 (10)

**Table 2:** Yeast genera identified in OSCC and control samples based on MALDI-TOF–MS analysis

Genus	No. of isolates (%)	
	Control (*N* = 12)	OSCC (*N* = 22)
*Candida*	10 (83.3)	15 (68.2)
*Rhodotorula*	–	2 (9.1)
*Saccharomyces*	–	2 (9.1)
*Kloeckera*	–	1 (4.5)
Other yeast (unidentified)	2 (16.7)	2 (9.1)

**Table 3:** Extracellular lipase/protease activity of *Candida* strains isolated from OSCC or control patients

No. of isolates (%)	lip+	lip−	prot+	prot−	lip+/prot+	lip+/prot−	lip−/prot+	lip−/prot−
Control (*N* = 40)								
*N*	13	27	15	25	6	7	9	18
%	32.5	67.5	37.5	62.5	15	17.5	22.5	45
OSCC (*N* = 140)								
*N*	53	87	66	74	37	16	29	58
%	37.86	62.14	47.14	52.86	26.43	11.43	20.71	41.43

## Discussion

*Candida* infection has long been implicated in cancer development, particularly in malignancies affecting the oral epithelium (Mohd et al. 2010). In this study, we found that the level of oral yeast carriage is significantly higher in patients with OSCC. It has been previously shown that there is positive association between oral yeast carriage and epithelial carcinoma (Nagy et al. 1998; Barrett et al. 1998; McCullough et al. 2002; Jahanshahi and Shirani 2015; Alnuaimi et al. 2015). Although the underlying mechanisms of how *Candida* species might promote carcinogenesis are still obscure, multiple mechanisms have been suggested to contribute to cancer development. These include the secretion of potential carcinogens, conversion of procarcinogens, damage of epithelial barriers by secreted proteolytic enzymes as well as the ability to induce chronic inflammation (Mohd et al. 2010). In a mouse model of carcinogensis, *C. albicans* infection has been shown to promote the development of 4 nitroquinoline 1-oxide (4NQO)-induced oral epithelial dysplasia, although the infection alone led only to the formation of hyperplastic lesions (Dwivedi et al. 2009). Here we tested a large number of yeast isolates from OSCC and control patients for extracellular enzyme production and found that there is no association between the lipase/protease producing capacity of strains and the higher colonisation rate of neoplastic epithelium. This finding questions the role of fungal hydrolytic enzymes in the development of epithelial dysplasia, although further experiments are needed to thoroughly examine their effect on carcinogenesis. It has also been suggested that *Candida* infection is the consequence rather than the cause of malignant oral disorders, as the neoplastic epithelium represents an immunosuppressive microenvironment that may promote the survival of *Candida* spp. It has been recognized during the recent years that there is a delicate interplay between the innate immune system and commensal fungi that is responsible for maintaining the integrity of the mucosa. On the one hand, components of the innate immune system recognize the invading pathogens and limit the overgrowth of commensal fungi by intra- or extracellular killing (Romani 2011). On the other hand, contact with commensal microbes induces tolerance, that limits host damage caused by excessive inflammation (Romani 2011). Interleukin-10 (IL-10) is one of the most effective mediators of immune tolerance and it has been shown that patients with chronic candidal diseases often present high levels of this cytokine (Romani 2011). It has been shown that patients with OSCC have increased levels of salivary IL-10 and high expression of this cytokine in tumor cells has been associated with poor prognosis (Chen et al. 2013; Aziz et al. 2015). Therefore, the antiinflammatory environment of neoplastic epithelium might support the proliferation of commensal yeasts by suppressing the activity of innate immune cells that are responsible for the limitation of microbial overgrowth. Using MALDI-TOF-MS analysis that represents a new and rapid method for the identification of yeasts in clinical samples (Pinto et al. 2011; Bader et al. 2011), we found that in addition to higher fungal burdens, the spectrum of isolated yeast genera was wider in samples derived from OSCC patients compared to healthy controls. This finding further strengthens the notion that the altered microenvironment associated with tumorigenesis leads to the development of a more diverse oral microflora. In conclusion, our results reveal important differences in the oral fungal flora of OSCC patients compared to healthy individuals, although further research is needed to elucidate the etiologic relationship between *Candida* infection and carcinogenesis.
